# Supplementary material for: Spatial and temporal shifts in the diet of the barnacle Amphibalanus eburneus within a subtropical estuary
Source: PeerJ. 2018 Aug 15;6:e5485. doi: 10.7717/peerj.5485 (PMC6098678; doi:10.7717/peerj.5485)
Supplement: Supplemental Information 2 [file peerj-06-5485-s002.pdf]

Site	Habitat	GPS-Latitude N	GPS-Longitude W
Banana 1 (B1)	Seawall	28°30.816	80°36.736
Banana 2 (B2)	Mangrove	28°27.875	80°37.861
Banana 2alt (B2a)	Mangrove	28°29.260	80°37.238
Banana 4 (B4)	Dock piling	28°21.434	80°37.711
Banana 5 (B5)	Mangrove	28°20.052	80°40.175
Banana 6 (B6)	Mangrove	28°18.639	80°37.094
Banana 6alt (B6a)	Mangrove	28°17.837	80°37.611
Banana 7 (B7)	Dock piling	28°17.001	80°36.613
Banana 8 (B8)	Seawall	28°12.619	80°37.599
Banana 9 (B9)	Shallow pilings	28°10.441	80.37.234
Indian River 1 (I1)	Mangrove	28°42.557	80°43.989
Indian River 2 (I2)	Bridge pilings	28°37.321	80°47.753
Indian River 3 (I3)	Wooden breakwater	28°33.246	80°47.669
Indian River 4 (I4)	Bridge pilings	28°31.641	80°46.060
Indian River 5 (I5)	Mangrove	28°28.775	80°43.548
Indian River 6 (I6)	Pilings	28°28.414	80°45.588
Indian River 7 (I7)	Seawall	28°24.119	80°44.289
Indian River 8 (I8)	Piling	28°21.297	80°43.333
Indian River 9 (I9)	Mangrove	28°20.033	80°42.178
Indian River 10 (I10)	Mangrove	28°19.468	80°41.997
Indian River 11 (I11)	Piling	28°18.046	80°41.782
Indian River 12 (I12)	Mangrove	28°13.077	80°38.773
Indian River 13d (I13)	Dock/piling	28°16.496	80°40.399
Indian River 13m (I13m)	Mangrove	28°16.505	80°40.394
Mosquito 1 (M1)	Dock/piling	28°53.845	80°51.075
Mosquito 2 (M2)	Dock/piling	28°52.388	80°50.291
Mosquito 4 (M4)	Manatee pilings	28°45.463	80°45.753
Mosquito 5 (M5)	Warning markers	28°43.479	80°46.065
Mosquito 6 (M6)	Wooden piling	28°43.917	80°43.044
