# Supplementary figures and images for: Spatial and temporal shifts in the diet of the barnacle Amphibalanus eburneus within a subtropical estuary
Source: PeerJ. 2018 Aug 15;6:e5485. doi: 10.7717/peerj.5485 (PMC6098678; doi:10.7717/peerj.5485)

2014

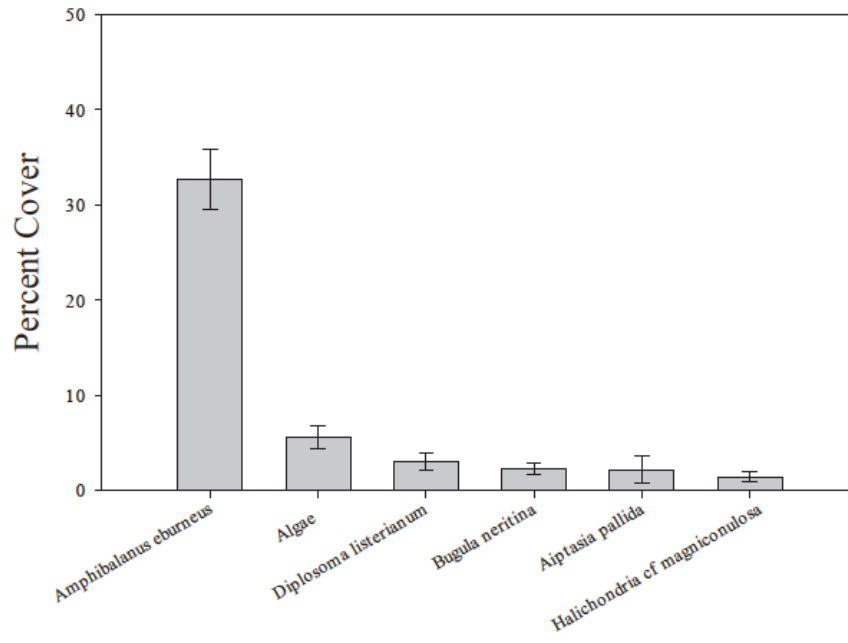

2016

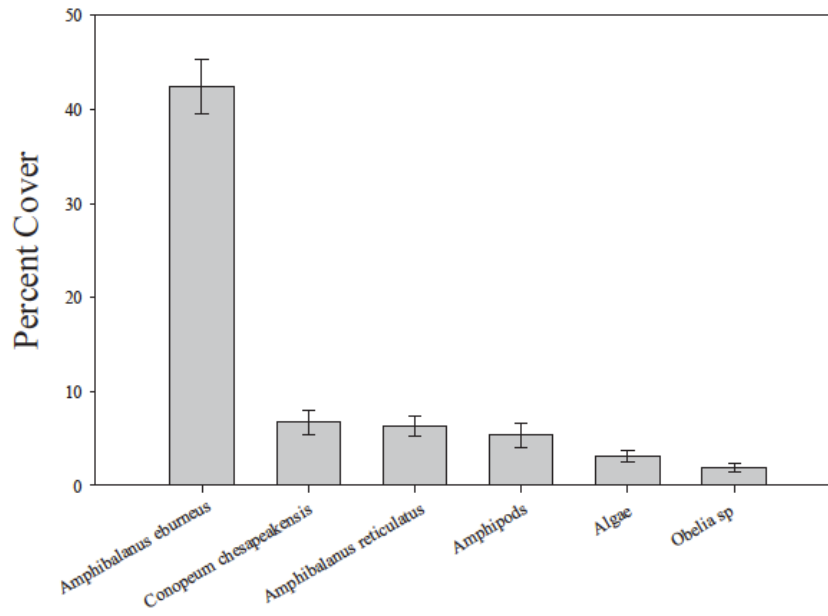

Supplement: Supplemental Information 3 — The same 90 sites were surveyed both times. [file peerj-06-5485-s003.pdf]

**Average Weekly Chlorophyll Levels**

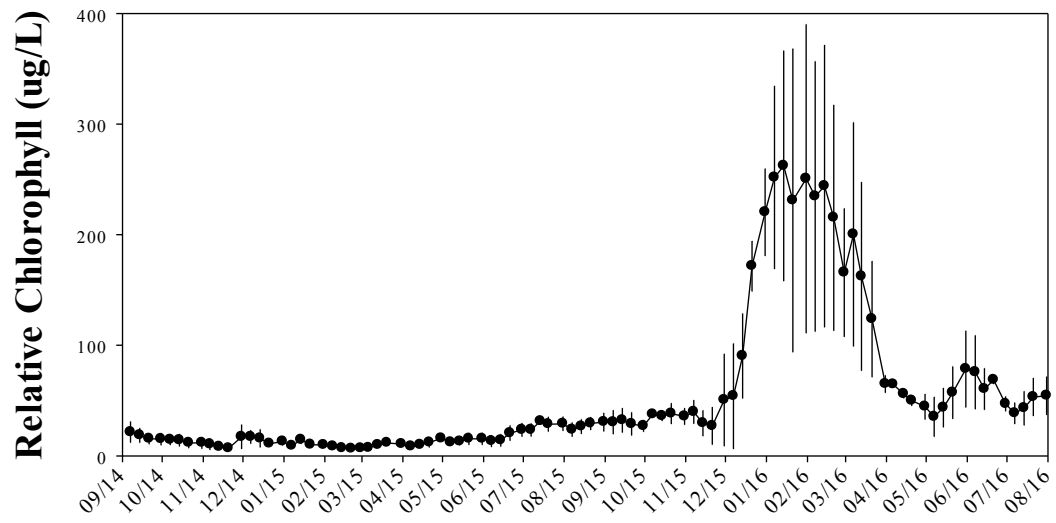

Supplement: Supplemental Information 4 — Data were derived from St. Johns River Water Management District: http://webapub.sjrwmd.com/agws10/hdswq/. [file peerj-06-5485-s004.pdf]

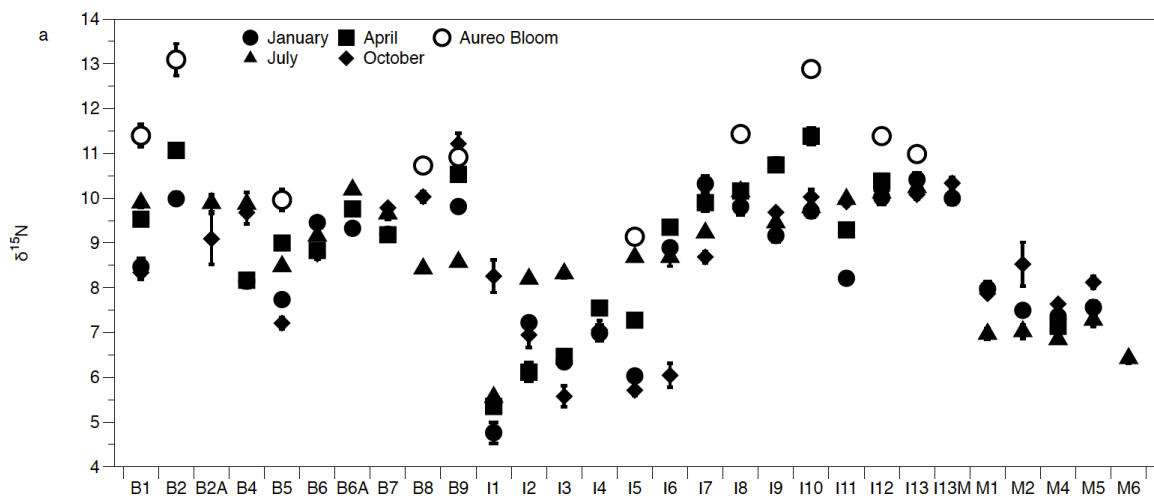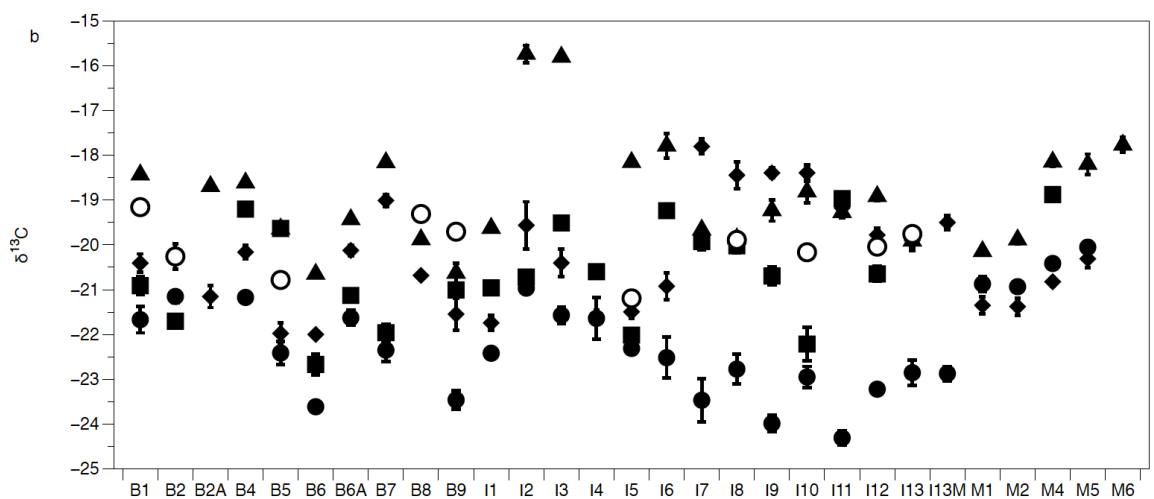

Supplement: Supplemental Information 5 — Sites were within the sub-lagoons of the North Indian River Lagoon (Banana River [B], Indian River Lagoon [I], and Mosquito Lagoon [M]). Data are shown for each season from January to October of 2015 and during an Aureoumbra lagunensis bloom in the January of 2016. [file peerj-06-5485-s005.pdf]

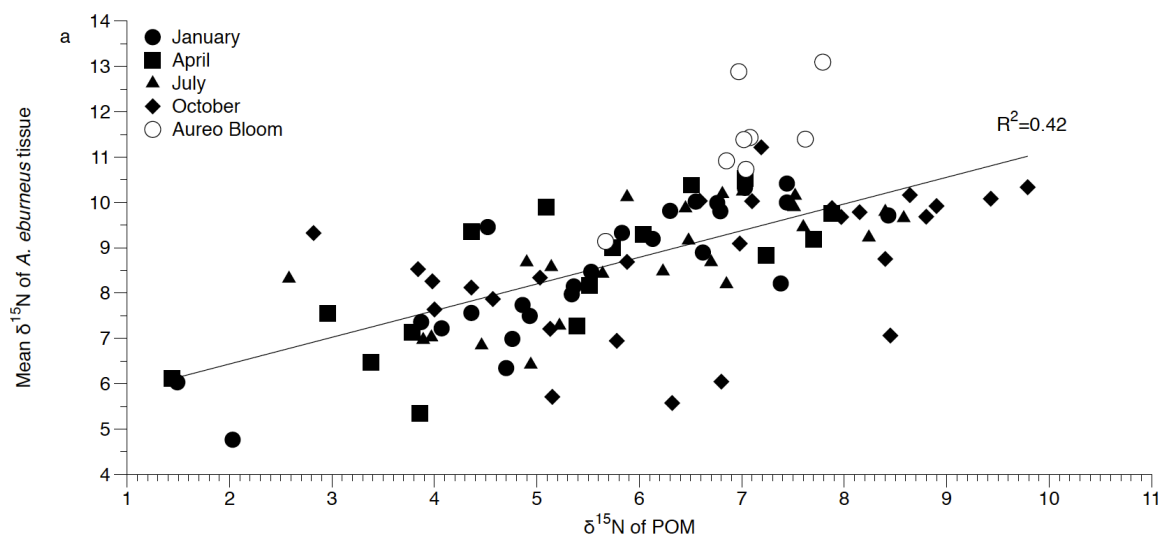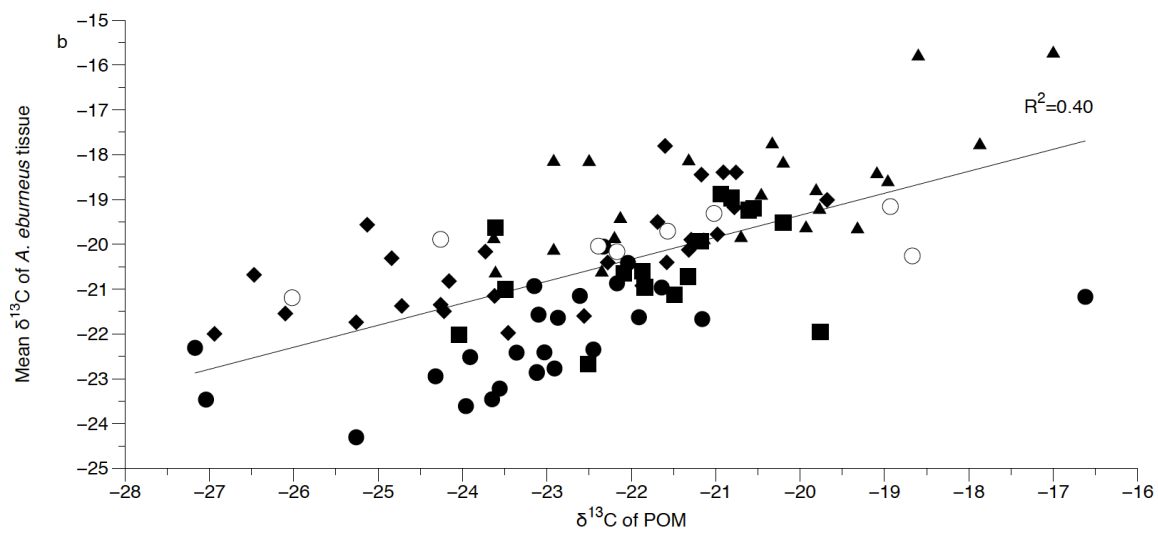

Supplement: Supplemental Information 6 — Data include 29 sites within the sublagoons of the North Indian River Lagoon. Tissue and POM samples were taken each season from January to October of 2015 and during an Aureoumbra lagunensis bloom in the January of 2016. [file peerj-06-5485-s006.pdf]
